# Supplementary material for: Highly pathogenic avian influenza A(H5N1) virus in a common bottlenose dolphin (Tursiops truncatus) in Florida
Source: Commun Biol. 2024 Apr 18;7:476. doi: 10.1038/s42003-024-06173-x (PMC11026403; doi:10.1038/s42003-024-06173-x)
Supplement: Supplementary file 2 — Supplementary Information [file 42003_2024_6173_MOESM2_ESM.pdf]

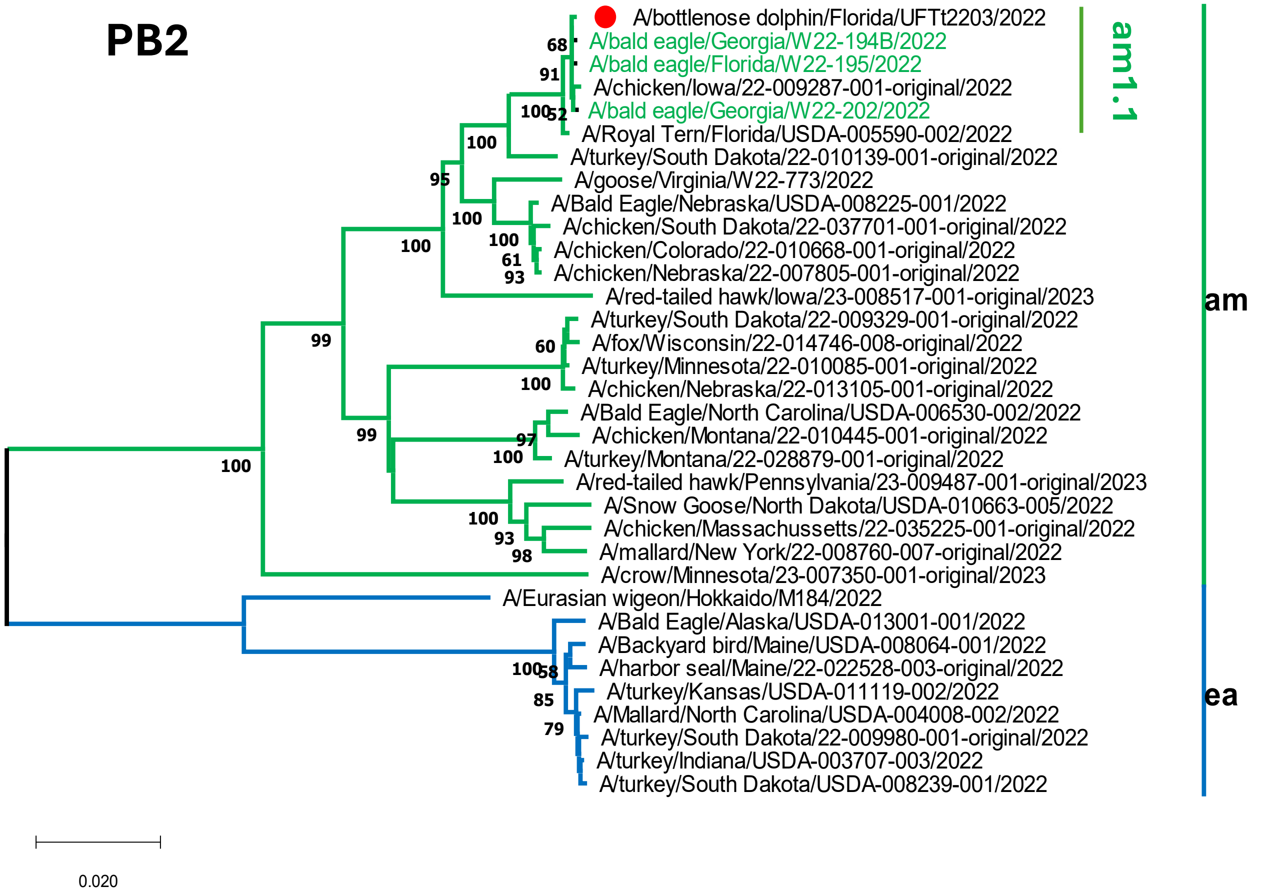
PB2
A/bottlenose dolphin/Florida/UFTt2203/2022
A/bald eagle/Georgia/W22-194B/2022
A/bald eagle/Florida/W22-195/2022
A/chicken/Iowa/22-009287-001-original/2022
A/bald eagle/Georgia/W22-202/2022
A/Royal Tern/Florida/USDA-005590-002/2022
A/turkey/South Dakota/22-010139-001-original/2022
A/goose/Virginia/W22-773/2022
A/Bald Eagle/Nebraska/USDA-008225-001/2022
A/chicken/South Dakota/22-037701-001-original/2022
A/chicken/Colorado/22-010668-001-original/2022
A/chicken/Nebraska/22-007805-001-original/2022
A/red-tailed hawk/Iowa/23-008517-001-original/2023
A/turkey/South Dakota/22-009329-001-original/2022
A/fox/Wisconsin/22-014746-008-original/2022
A/turkey/Minnesota/22-010085-001-original/2022
A/chicken/Nebraska/22-013105-001-original/2022
A/Bald Eagle/North Carolina/USDA-006530-002/2022
A/chicken/Montana/22-010445-001-original/2022
A/turkey/Montana/22-028879-001-original/2022
A/red-tailed hawk/Pennsylvania/23-009487-001-original/2023
A/Snow Goose/North Dakota/USDA-010663-005/2022
A/chicken/Massachussetts/22-035225-001-original/2022
A/mallard/New York/22-008760-007-original/2022
A/crow/Minnesota/23-007350-001-original/2023
A/Eurasian wigeon/Hokkaido/M184/2022
A/Bald Eagle/Alaska/USDA-013001-001/2022
A/Backyard bird/Maine/USDA-008064-001/2022
A/harbor seal/Maine/22-022528-003-original/2022
A/turkey/Kansas/USDA-011119-002/2022
A/Mallard/North Carolina/USDA-004008-002/2022
A/turkey/South Dakota/22-009980-001-original/2022
A/turkey/Indiana/USDA-003707-003/2022
A/turkey/South Dakota/USDA-008239-001/2022
68 91 100 52 100 95 100 100 100 61 93 99 60 100 99 97 100 100 93 98 100 100 58 85 79
am1.1
am
ea
0.020

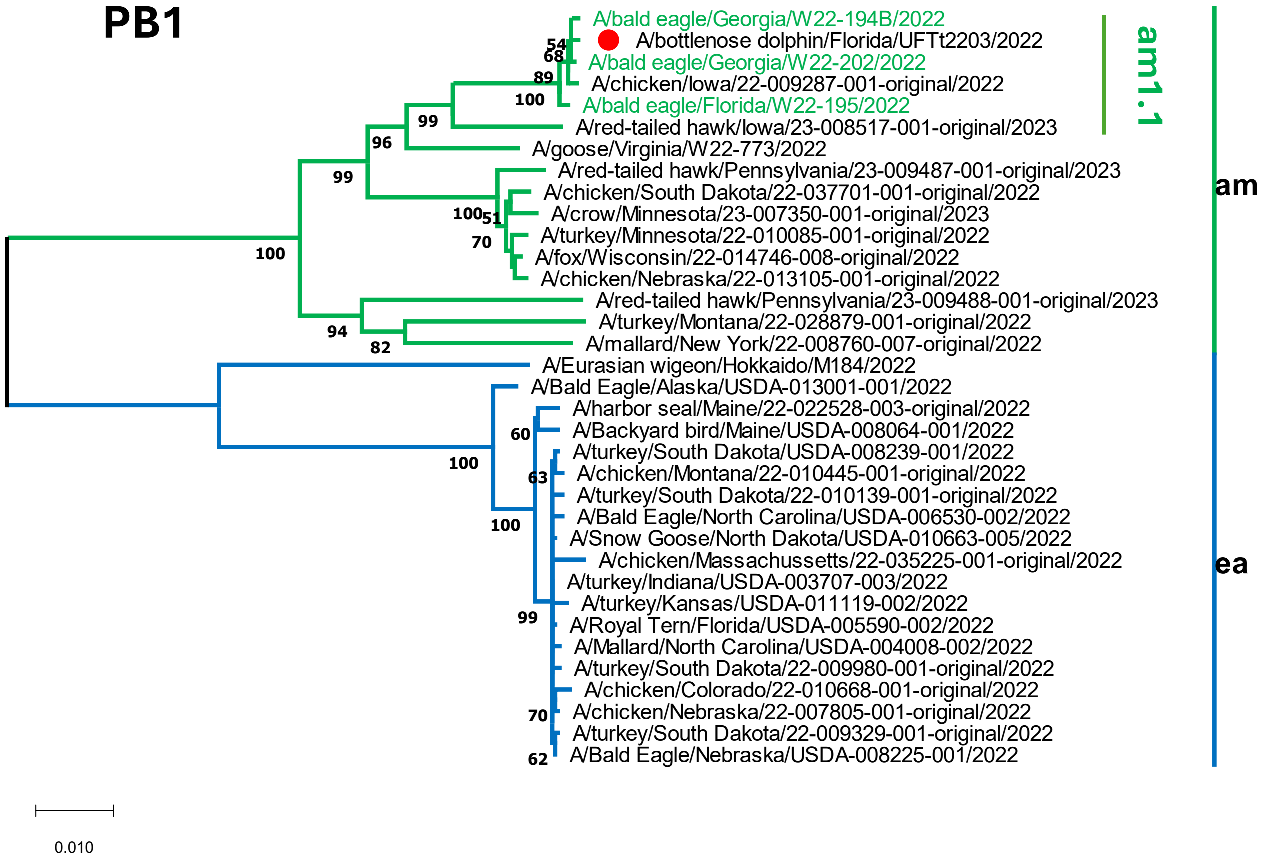
PB1
A/bald eagle/Georgia/W22-194B/2022
A/bottlenose dolphin/Florida/UFTt2203/2022
A/bald eagle/Georgia/W22-202/2022
A/chicken/Iowa/22-009287-001-original/2022
A/bald eagle/Florida/W22-195/2022
A/red-tailed hawk/Iowa/23-008517-001-original/2023
A/goose/Virginia/W22-773/2022
A/red-tailed hawk/Pennsylvania/23-009487-001-original/2023
A/chicken/South Dakota/22-037701-001-original/2022
A/crow/Minnesota/23-007350-001-original/2023
A/turkey/Minnesota/22-010085-001-original/2022
A/fox/Wisconsin/22-014746-008-original/2022
A/chicken/Nebraska/22-013105-001-original/2022
A/red-tailed hawk/Pennsylvania/23-009488-001-original/2023
A/turkey/Montana/22-028879-001-original/2022
A/mallard/New York/22-008760-007-original/2022
A/Eurasian wigeon/Hokkaido/M184/2022
A/Bald Eagle/Alaska/USDA-013001-001/2022
A/harbor seal/Maine/22-022528-003-original/2022
A/Backyard bird/Maine/USDA-008064-001/2022
A/turkey/South Dakota/USDA-008239-001/2022
A/chicken/Montana/22-010445-001-original/2022
A/turkey/South Dakota/22-010139-001-original/2022
A/Bald Eagle/North Carolina/USDA-006530-002/2022
A/Snow Goose/North Dakota/USDA-010663-005/2022
A/chicken/Massachussetts/22-035225-001-original/2022
A/turkey/Indiana/USDA-003707-003/2022
A/turkey/Kansas/USDA-011119-002/2022
A/Royal Tern/Florida/USDA-005590-002/2022
A/Mallard/North Carolina/USDA-004008-002/2022
A/turkey/South Dakota/22-009980-001-original/2022
A/chicken/Colorado/22-010668-001-original/2022
A/chicken/Nebraska/22-007805-001-original/2022
A/turkey/South Dakota/22-009329-001-original/2022
A/Bald Eagle/Nebraska/USDA-008225-001/2022
54 68 89 100 99 96 99 100 51 70 100 94 82 60 100 63 100 99 70 62
am1.1
am
ea
0.010

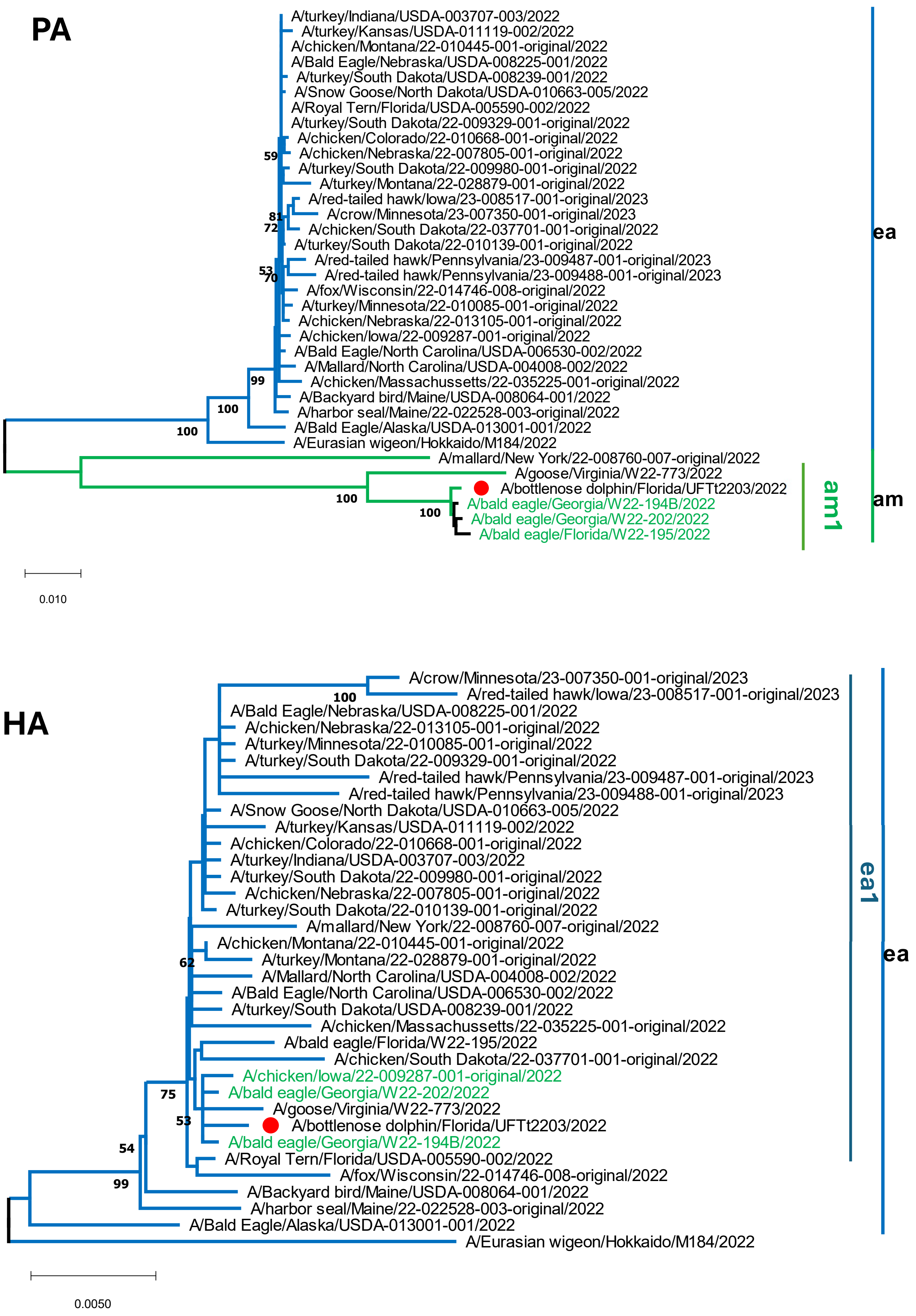
PA
A/turkey/Indiana/USDA-003707-003/2022
A/turkey/Kansas/USDA-011119-002/2022
A/chicken/Montana/22-010445-001-original/2022
A/Bald Eagle/Nebraska/USDA-008225-001/2022
A/turkey/South Dakota/USDA-008239-001/2022
A/Snow Goose/North Dakota/USDA-010663-005/2022
A/Royal Tern/Florida/USDA-005590-002/2022
A/turkey/South Dakota/22-009329-001-original/2022
A/chicken/Colorado/22-010668-001-original/2022
A/chicken/Nebraska/22-007805-001-original/2022
A/turkey/South Dakota/22-009980-001-original/2022
A/turkey/Montana/22-028879-001-original/2022
A/red-tailed hawk/Iowa/23-008517-001-original/2023
A/crow/Minnesota/23-007350-001-original/2023
A/chicken/South Dakota/22-037701-001-original/2022
A/turkey/South Dakota/22-010139-001-original/2022
A/red-tailed hawk/Pennsylvania/23-009487-001-original/2023
A/red-tailed hawk/Pennsylvania/23-009488-001-original/2023
A/fox/Wisconsin/22-014746-008-original/2022
A/turkey/Minnesota/22-010085-001-original/2022
A/chicken/Nebraska/22-013105-001-original/2022
A/chicken/Iowa/22-009287-001-original/2022
A/Bald Eagle/North Carolina/USDA-006530-002/2022
A/Mallard/North Carolina/USDA-004008-002/2022
A/chicken/Massachussetts/22-035225-001-original/2022
A/Backyard bird/Maine/USDA-008064-001/2022
A/harbor seal/Maine/22-022528-003-original/2022
A/Bald Eagle/Alaska/USDA-013001-001/2022
A/Eurasian wigeon/Hokkaido/M184/2022
A/mallard/New York/22-008760-007-original/2022
A/goose/Virginia/W22-773/2022
A/bottlenose dolphin/Florida/UFTt2203/2022
A/bald eagle/Georgia/W22-194B/2022
A/bald eagle/Georgia/W22-202/2022
A/bald eagle/Florida/W22-195/2022
59
81
72
53
70
99
100
100
100
100
ea
am1
am
0.010
HA
A/crow/Minnesota/23-007350-001-original/2023
A/red-tailed hawk/Iowa/23-008517-001-original/2023
100
A/Bald Eagle/Nebraska/USDA-008225-001/2022
A/chicken/Nebraska/22-013105-001-original/2022
A/turkey/Minnesota/22-010085-001-original/2022
A/turkey/South Dakota/22-009329-001-original/2022
A/red-tailed hawk/Pennsylvania/23-009487-001-original/2023
A/red-tailed hawk/Pennsylvania/23-009488-001-original/2023
A/Snow Goose/North Dakota/USDA-010663-005/2022
A/turkey/Kansas/USDA-011119-002/2022
A/chicken/Colorado/22-010668-001-original/2022
A/turkey/Indiana/USDA-003707-003/2022
A/turkey/South Dakota/22-009980-001-original/2022
A/chicken/Nebraska/22-007805-001-original/2022
A/turkey/South Dakota/22-010139-001-original/2022
A/mallard/New York/22-008760-007-original/2022
A/chicken/Montana/22-010445-001-original/2022
A/turkey/Montana/22-028879-001-original/2022
A/Mallard/North Carolina/USDA-004008-002/2022
A/Bald Eagle/North Carolina/USDA-006530-002/2022
A/turkey/South Dakota/USDA-008239-001/2022
A/chicken/Massachussetts/22-035225-001-original/2022
A/bald eagle/Florida/W22-195/2022
A/chicken/South Dakota/22-037701-001-original/2022
A/chicken/Iowa/22-009287-001-original/2022
A/bald eagle/Georgia/W22-202/2022
A/goose/Virginia/W22-773/2022
A/bottlenose dolphin/Florida/UFTt2203/2022
A/bald eagle/Georgia/W22-194B/2022
A/Royal Tern/Florida/USDA-005590-002/2022
A/fox/Wisconsin/22-014746-008-original/2022
A/Backyard bird/Maine/USDA-008064-001/2022
A/harbor seal/Maine/22-022528-003-original/2022
A/Bald Eagle/Alaska/USDA-013001-001/2022
A/Eurasian wigeon/Hokkaido/M184/2022
62
75
53
54
99
ea1
ea
0.0050

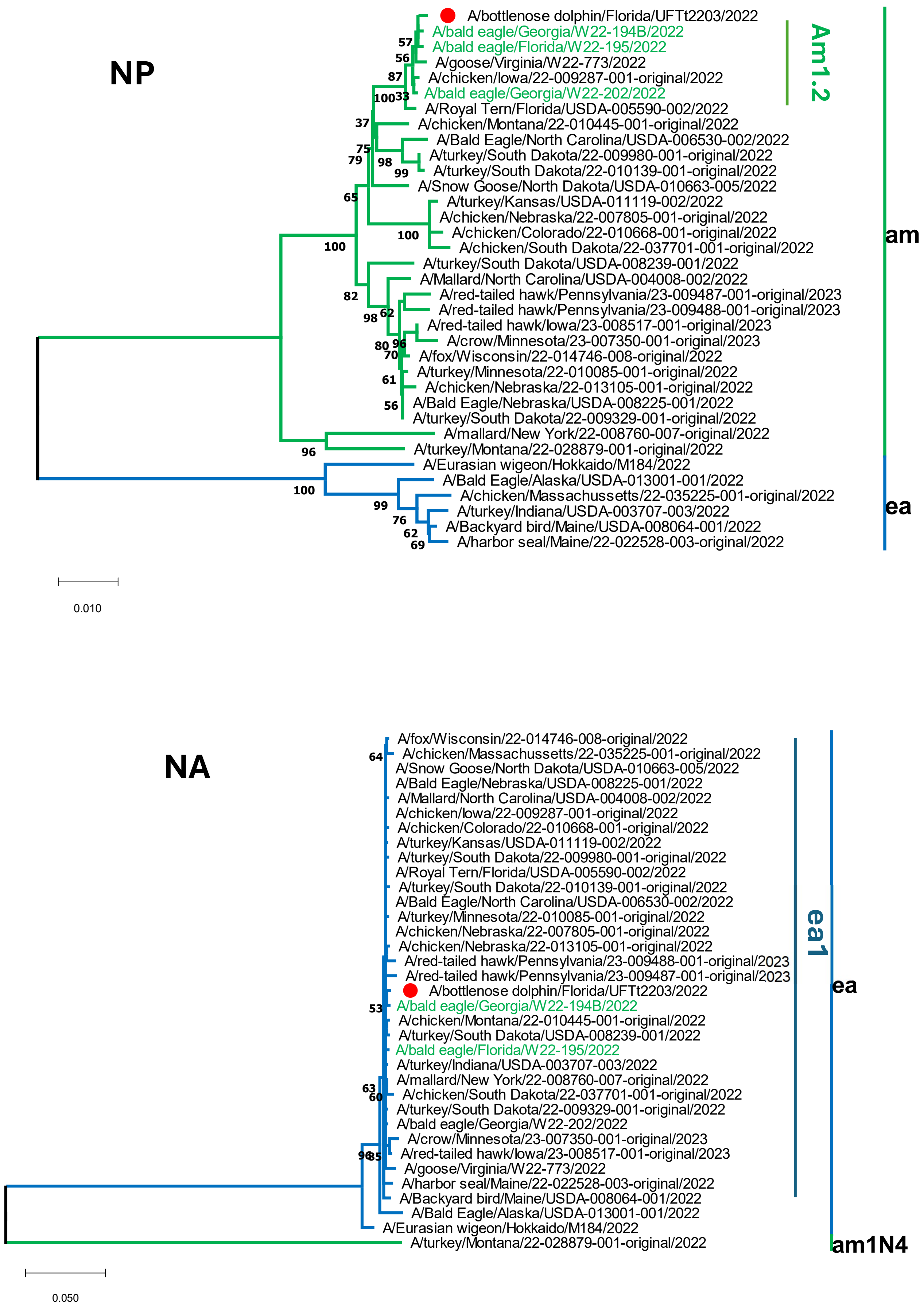
NP
A/bottlenose dolphin/Florida/UFTt2203/2022
A/bald eagle/Georgia/W22-194B/2022
A/bald eagle/Florida/W22-195/2022
A/goose/Virginia/W22-773/2022
A/chicken/Iowa/22-009287-001-original/2022
A/bald eagle/Georgia/W22-202/2022
A/Royal Tern/Florida/USDA-005590-002/2022
A/chicken/Montana/22-010445-001-original/2022
A/Bald Eagle/North Carolina/USDA-006530-002/2022
A/turkey/South Dakota/22-009980-001-original/2022
A/turkey/South Dakota/22-010139-001-original/2022
A/Snow Goose/North Dakota/USDA-010663-005/2022
A/turkey/Kansas/USDA-011119-002/2022
A/chicken/Nebraska/22-007805-001-original/2022
A/chicken/Colorado/22-010668-001-original/2022
A/chicken/South Dakota/22-037701-001-original/2022
A/turkey/South Dakota/USDA-008239-001/2022
A/Mallard/North Carolina/USDA-004008-002/2022
A/red-tailed hawk/Pennsylvania/23-009487-001-original/2023
A/red-tailed hawk/Pennsylvania/23-009488-001-original/2023
A/red-tailed hawk/Iowa/23-008517-001-original/2023
A/crow/Minnesota/23-007350-001-original/2023
A/fox/Wisconsin/22-014746-008-original/2022
A/turkey/Minnesota/22-010085-001-original/2022
A/chicken/Nebraska/22-013105-001-original/2022
A/Bald Eagle/Nebraska/USDA-008225-001/2022
A/turkey/South Dakota/22-009329-001-original/2022
A/mallard/New York/22-008760-007-original/2022
A/turkey/Montana/22-028879-001-original/2022
A/Eurasian wigeon/Hokkaido/M184/2022
A/Bald Eagle/Alaska/USDA-013001-001/2022
A/chicken/Massachussetts/22-035225-001-original/2022
A/turkey/Indiana/USDA-003707-003/2022
A/Backyard bird/Maine/USDA-008064-001/2022
A/harbor seal/Maine/22-022528-003-original/2022
Am1.2
am
ea
0.010
NA
A/fox/Wisconsin/22-014746-008-original/2022
A/chicken/Massachussetts/22-035225-001-original/2022
A/Snow Goose/North Dakota/USDA-010663-005/2022
A/Bald Eagle/Nebraska/USDA-008225-001/2022
A/Mallard/North Carolina/USDA-004008-002/2022
A/chicken/Iowa/22-009287-001-original/2022
A/chicken/Colorado/22-010668-001-original/2022
A/turkey/Kansas/USDA-011119-002/2022
A/turkey/South Dakota/22-009980-001-original/2022
A/Royal Tern/Florida/USDA-005590-002/2022
A/turkey/South Dakota/22-010139-001-original/2022
A/Bald Eagle/North Carolina/USDA-006530-002/2022
A/turkey/Minnesota/22-010085-001-original/2022
A/chicken/Nebraska/22-007805-001-original/2022
A/chicken/Nebraska/22-013105-001-original/2022
A/red-tailed hawk/Pennsylvania/23-009488-001-original/2023
A/red-tailed hawk/Pennsylvania/23-009487-001-original/2023
A/bottlenose dolphin/Florida/UFTt2203/2022
A/bald eagle/Georgia/W22-194B/2022
A/chicken/Montana/22-010445-001-original/2022
A/turkey/South Dakota/USDA-008239-001/2022
A/bald eagle/Florida/W22-195/2022
A/turkey/Indiana/USDA-003707-003/2022
A/mallard/New York/22-008760-007-original/2022
A/chicken/South Dakota/22-037701-001-original/2022
A/turkey/South Dakota/22-009329-001-original/2022
A/bald eagle/Georgia/W22-202/2022
A/crow/Minnesota/23-007350-001-original/2023
A/red-tailed hawk/Iowa/23-008517-001-original/2023
A/goose/Virginia/W22-773/2022
A/harbor seal/Maine/22-022528-003-original/2022
A/Backyard bird/Maine/USDA-008064-001/2022
A/Bald Eagle/Alaska/USDA-013001-001/2022
A/Eurasian wigeon/Hokkaido/M184/2022
A/turkey/Montana/22-028879-001-original/2022
ea1
ea
am1N4
0.050

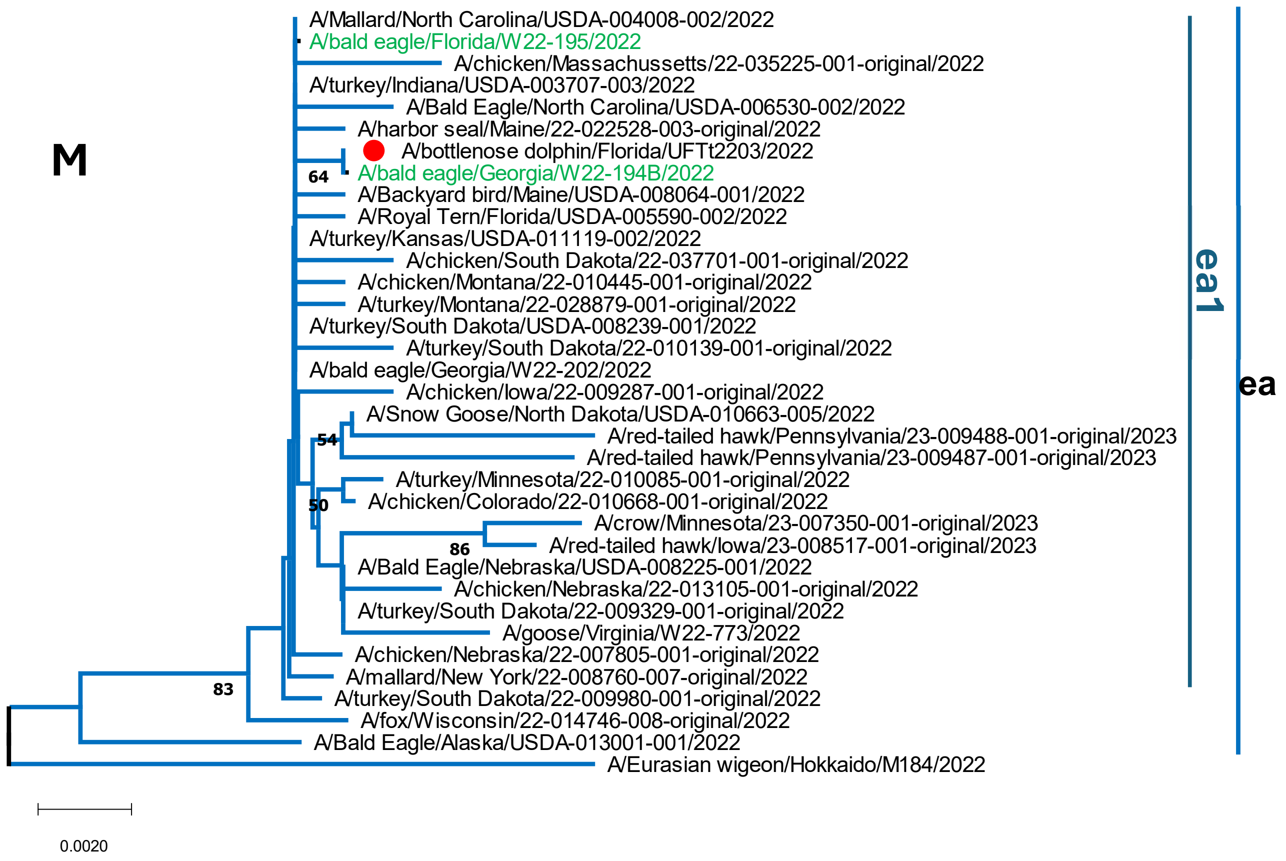
M
A/Mallard/North Carolina/USDA-004008-002/2022
A/bald eagle/Florida/W22-195/2022
A/chicken/Massachussetts/22-035225-001-original/2022
A/turkey/Indiana/USDA-003707-003/2022
A/Bald Eagle/North Carolina/USDA-006530-002/2022
A/harbor seal/Maine/22-022528-003-original/2022
A/bottlenose dolphin/Florida/UFTt2203/2022
64
A/bald eagle/Georgia/W22-194B/2022
A/Backyard bird/Maine/USDA-008064-001/2022
A/Royal Tern/Florida/USDA-005590-002/2022
A/turkey/Kansas/USDA-011119-002/2022
A/chicken/South Dakota/22-037701-001-original/2022
A/chicken/Montana/22-010445-001-original/2022
A/turkey/Montana/22-028879-001-original/2022
A/turkey/South Dakota/USDA-008239-001/2022
A/turkey/South Dakota/22-010139-001-original/2022
A/bald eagle/Georgia/W22-202/2022
A/chicken/Iowa/22-009287-001-original/2022
A/Snow Goose/North Dakota/USDA-010663-005/2022
54
A/red-tailed hawk/Pennsylvania/23-009488-001-original/2023
A/red-tailed hawk/Pennsylvania/23-009487-001-original/2023
A/turkey/Minnesota/22-010085-001-original/2022
50
A/chicken/Colorado/22-010668-001-original/2022
A/crow/Minnesota/23-007350-001-original/2023
86
A/red-tailed hawk/Iowa/23-008517-001-original/2023
A/Bald Eagle/Nebraska/USDA-008225-001/2022
A/chicken/Nebraska/22-013105-001-original/2022
A/turkey/South Dakota/22-009329-001-original/2022
A/goose/Virginia/W22-773/2022
A/chicken/Nebraska/22-007805-001-original/2022
83
A/mallard/New York/22-008760-007-original/2022
A/turkey/South Dakota/22-009980-001-original/2022
A/fox/Wisconsin/22-014746-008-original/2022
A/Bald Eagle/Alaska/USDA-013001-001/2022
A/Eurasian wigeon/Hokkaido/M184/2022
ea1
ea
0.0020

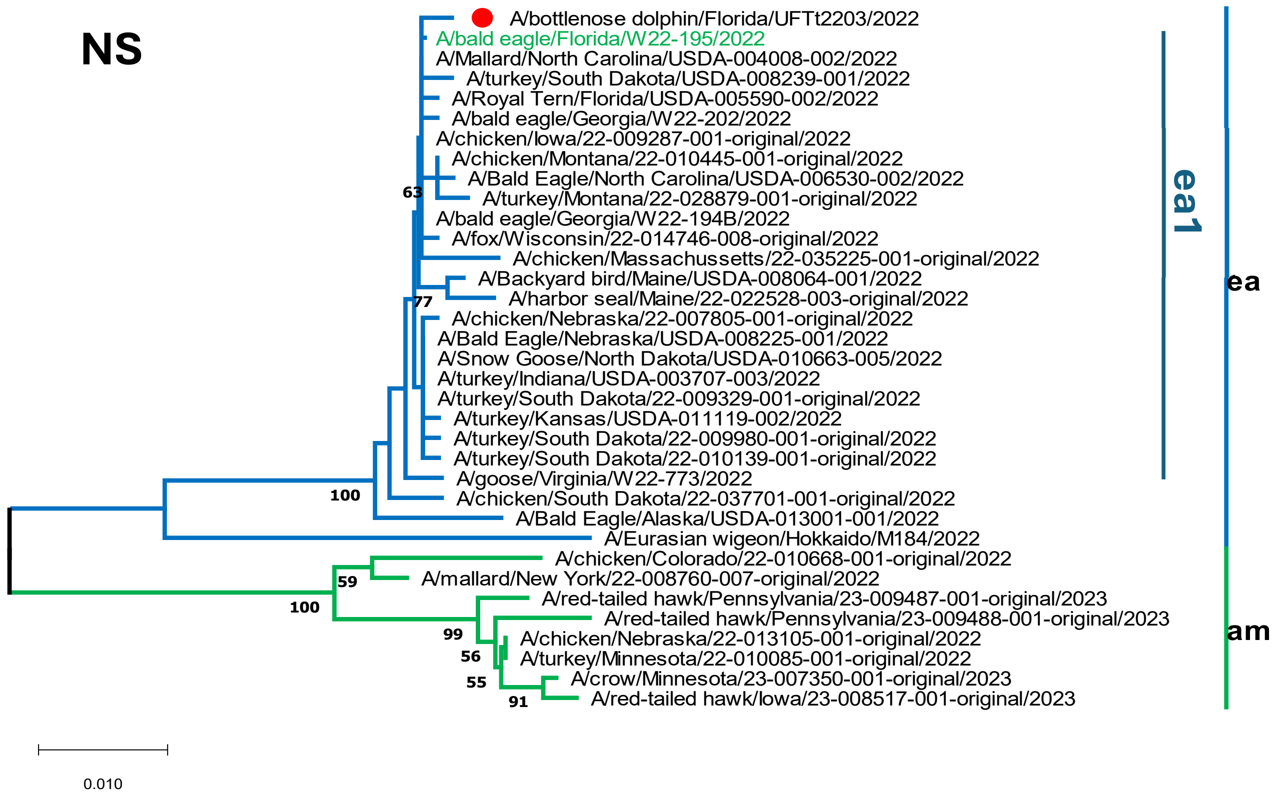
NS
A/bottlenose dolphin/Florida/UFTt2203/2022
A/bald eagle/Florida/W22-195/2022
A/Mallard/North Carolina/USDA-004008-002/2022
A/turkey/South Dakota/USDA-008239-001/2022
A/Royal Tern/Florida/USDA-005590-002/2022
A/bald eagle/Georgia/W22-202/2022
A/chicken/Iowa/22-009287-001-original/2022
A/chicken/Montana/22-010445-001-original/2022
A/Bald Eagle/North Carolina/USDA-006530-002/2022
63
A/turkey/Montana/22-028879-001-original/2022
A/bald eagle/Georgia/W22-194B/2022
A/fox/Wisconsin/22-014746-008-original/2022
A/chicken/Massachussetts/22-035225-001-original/2022
A/Backyard bird/Maine/USDA-008064-001/2022
77
A/harbor seal/Maine/22-022528-003-original/2022
A/chicken/Nebraska/22-007805-001-original/2022
A/Bald Eagle/Nebraska/USDA-008225-001/2022
A/Snow Goose/North Dakota/USDA-010663-005/2022
A/turkey/Indiana/USDA-003707-003/2022
A/turkey/South Dakota/22-009329-001-original/2022
A/turkey/Kansas/USDA-011119-002/2022
A/turkey/South Dakota/22-009980-001-original/2022
A/turkey/South Dakota/22-010139-001-original/2022
A/goose/Virginia/W22-773/2022
100
A/chicken/South Dakota/22-037701-001-original/2022
A/Bald Eagle/Alaska/USDA-013001-001/2022
A/Eurasian wigeon/Hokkaido/M184/2022
A/chicken/Colorado/22-010668-001-original/2022
59
A/mallard/New York/22-008760-007-original/2022
100
A/red-tailed hawk/Pennsylvania/23-009487-001-original/2023
A/red-tailed hawk/Pennsylvania/23-009488-001-original/2023
99
A/chicken/Nebraska/22-013105-001-original/2022
56
A/turkey/Minnesota/22-010085-001-original/2022
55
A/crow/Minnesota/23-007350-001-original/2023
91
A/red-tailed hawk/Iowa/23-008517-001-original/2023
ea1
ea
am
0.010

Fig. S1. Phylogenetic trees of eight gene segments of A/bottlenose dolphin/Florida/UFTt2203/2022 (H5N1). A/bottlenose dolphin/Florida/UFTt2203/2022 is indicated by the red circle and viruses with the highest sequence identity to this virus are in green font. am represents North American gene lineages, colored green; and ea representing Eurasian gene lineage are colored blue. The genetic clade to which A/bottlenose dolphin/Florida/UFTt2203/2022 belongs to is identified to the right. Numbers at nodes represent bootstrap percentages.

Table S1. GISAID Acknowledgement table. All sequences downloaded from GISAID utilized for phylogenetic analysis are represented and ordered by the GISAID Isolate ID number and including strain name, gene segment IDs and sequence authors.

We gratefully acknowledge the authors, originating and submitting laboratories of the sequences from GISAID's EpiFlu™ Database on which this research is based. The list is detailed below.			
Isolate-ID	Isolate name	Segment IDs	Authors
EPI_ISL_18133097	A/Backyard bird/Maine/USDA008064-001/2022	EPI2709682, EPI2709685, EPI2709688, EPI2709691, EPI2709694, EPI2709697, EPI2709670, EPI2709673	Youk,S., Torchetti,M.K., Lantz,K., Lenoch,J.B., Killian,M.L., Leyson,C., Bevins,S.N., Dilione,K., Ip,H.S., Stallknecht,D.E., Poulson,R.L., Suarez,D.L., Swayne,D.E.; PantinJackwood,M.J.
EPI_ISL_18132957	A/Bald Eagle/Alaska/USDA013001-001/2022	EPI2708558, EPI2708572, EPI2708545, EPI2708547, EPI2708556, EPI2708565, EPI2708567, EPI2708570	Youk,S., Torchetti,M.K., Lantz,K., Lenoch,J.B., Killian,M.L., Leyson,C., Bevins,S.N., Dilione,K., Ip,H.S., Stallknecht,D.E., Poulson,R.L., Suarez,D.L., Swayne,D.E.; PantinJackwood,M.J.
EPI_ISL_14937070	A/bald eagle/Florida/W22195/2022	EPI2176439, EPI2176440, EPI2176441, EPI2176442, EPI2176443, EPI2176444, EPI2176445, EPI2176446	Poulson,R.; Stallknecht,D.; Rubrum,A.; Jeevan,T.; Walker,D.; Webby,R.
EPI_ISL_14937002	A/bald eagle/Georgia/W22194B/2022	EPI2176424, EPI2176425, EPI2176426, EPI2176427, EPI2176428, EPI2176429, EPI2176430, EPI2176431	Poulson,R.; Stallknecht,D.; Rubrum,A.; Jeevan,T.; Walker,D.; Webby,R.
EPI_ISL_14937071	A/bald eagle/Georgia/W22202/2022	EPI2176449, EPI2176450, EPI2176451, EPI2176452, EPI2176453, EPI2176454, EPI2176447, EPI2176448	Poulson,R.; Stallknecht,D.; Rubrum,A.; Jeevan,T.; Walker,D.; Webby,R.
EPI_ISL_18132909	A/Bald Eagle/Nebraska/USDA008225-001/2022	EPI2708180, EPI2708182, EPI2708187, EPI2708190, EPI2708194, EPI2708197, EPI2708168, EPI2708172	Youk,S., Torchetti,M.K., Lantz,K., Lenoch,J.B., Killian,M.L., Leyson,C., Bevins,S.N., Dilione,K., Ip,H.S., Stallknecht,D.E., Poulson,R.L., Suarez,D.L., Swayne,D.E.; PantinJackwood,M.J.
EPI_ISL_18132885	A/Bald Eagle/North Carolina/USDA-006530-002/2022	EPI2707988, EPI2707990, EPI2707994, EPI2707997, EPI2707999, EPI2708004, EPI2707979, EPI2707981	Youk,S., Torchetti,M.K., Lantz,K., Lenoch,J.B., Killian,M.L., Leyson,C., Bevins,S.N., Dilione,K., Ip,H.S., Stallknecht,D.E., Poulson,R.L., Suarez,D.L., Swayne,D.E.; PantinJackwood,M.J.

EPI_ISL_15004062	A/chicken/Colorado/22-010668-001-original/2022	EPI2179297, EPI2179298, EPI2179293, EPI2179294, EPI2179295, EPI2179299, EPI2179300, EPI2179296	Chinh,Thanh;Franzen,Kerrie;Love, Emily;Killian,Mary;Koster,Leo;Lantz,Kristina;Stuber,Tod;Hicks,Jessica
EPI_ISL_12690589	A/chicken/Iowa/22-009287001-original/2022	EPI2035074, EPI2035075, EPI2035078, EPI2035079, EPI2035080, EPI2035076, EPI2035077, EPI2035081	Chinh,Thanh;Franzen,Kerrie;Love, Emily;Killian,Mary;Koster,Leo;Lantz,Kristina;Stuber,Tod;Hicks,Jessica
EPI_ISL_16297079	A/chicken/Massachussetts/22 -035225-001-original/2022	EPI2263542, EPI2263543, EPI2263544, EPI2263539, EPI2263540, EPI2263541, EPI2263545, EPI2263546	Chinh,Thanh;Love, Emily;Franzen,Kerrie;Killian,Mary;Koster,Leo;Lantz,Kristina;Stuber,Tod;Hicks,Jessica
EPI_ISL_13434407	A/chicken/Montana/22-010445-001-original/2022	EPI2074910, EPI2074911, EPI2074912, EPI2074913, EPI2074914, EPI2074915, EPI2074916, EPI2074909	Chinh,Thanh;Love, Emily;Franzen,Kerrie;Killian,Mary;Koster,Leo;Lantz,Kristina;Stuber,Tod;Hicks,Jessica
EPI_ISL_11897695	A/chicken/Nebraska/22-007805-001-original/2022	EPI2014121, EPI2014118, EPI2014119, EPI2014120, EPI2014122, EPI2014123, EPI2014124, EPI2014125	Chinh,Thanh;Love, Emily;Franzen,Kerrie;Killian,Mary;Koster,Leo;Lantz,Kristina;Stuber,Tod;Hicks,Jessica
EPI_ISL_16171473	A/chicken/Nebraska/22-013105-001-original/2022	EPI2246420, EPI2246421, EPI2246422, EPI2246417, EPI2246418, EPI2246419, EPI2246423, EPI2246424	Chinh,Thanh;Love, Emily;Franzen,Kerrie;Killian,Mary;Koster,Leo;Lantz,Kristina;Stuber,Tod;Hicks,Jessica
EPI_ISL_16271839	A/chicken/South Dakota/22037701-001-original/2022	EPI2260658, EPI2260659, EPI2260660, EPI2260655, EPI2260656, EPI2260657, EPI2260661, EPI2260662	Chinh,Thanh;Love, Emily;Franzen,Kerrie;Killian,Mary;Koster,Leo;Lantz,Kristina;Stuber,Tod;Hicks,Jessica
EPI_ISL_17964888	A/crow/Minnesota/23-007350-001-original/2023	EPI2613879, EPI2613880, EPI2613881, EPI2613882, EPI2613883, EPI2613884, EPI2613885, EPI2613886	Chinh,Thanh;Franzen,Kerrie;Love,Emily; Ozella,Mikaela;Killian,Mary;Lantz,Kristina;Stuber,Tod;Hicks,Jessica;Norris,Cameron
EPI_ISL_15732766	A/Eurasian wigeon/Hokkaido/M184/2022	EPI2213710, EPI2213711, EPI2213709, EPI2213713, EPI2213706, EPI2213708, EPI2213712, EPI2213707	Yoshihiro,SAKODA; Kohei,OGASAWARA; Norikazu,ISODA; Takahiro,HIONO
EPI_ISL_15078246	A/fox/Wisconsin/22-014746008-original/2022	EPI2182068, EPI2182069, EPI2182064, EPI2182065, EPI2182066, EPI2182067, EPI2182070, EPI2182071	Chinh,Thanh;Love, Emily;Franzen,Kerrie;Killian,Mary;Koster,Leo;Lantz,Kristina;Stuber,Tod;Hicks,Jessica
EPI_ISL_17424653	A/goose/Virginia/W22773/2022	EPI2507060, EPI2507061, EPI2507065, EPI2507063, EPI2507057, EPI2507059, EPI2507062, EPI2507064	Poulson,R.; Stallknecht,D.; Rubrum,A.; Jeevan,T.; Walker,D.; Webby,R.

EPI_ISL_16632539	A/harbor seal/Maine/22-022528-003-original/2022	EPI2310413, EPI2310414, EPI2310415, EPI2310416,	Chinh,Thanh; Franzen,Kerrie; Love,Emily; Killian,Mary; Lantz,Kristina; Hicks,Jessica; Norris, Cameron;
		EPI2310419, EPI2310420, EPI2310417, EPI2310418	Stuber,Tod; Sawatzki, Kaitlin; Puryear, Wendy; Foss, Alexa; Doughty,Linda; Runstadler, Jonathan
