# Supplementary material for: Highly pathogenic avian influenza A(H5N1) virus in a common bottlenose dolphin (Tursiops truncatus) in Florida
Source: Commun Biol. 2024 Apr 18;7:476. doi: 10.1038/s42003-024-06173-x (PMC11026403; doi:10.1038/s42003-024-06173-x)
Supplement: Supplementary file 3 — Description of Additional Supplementary Files [file 42003_2024_6173_MOESM3_ESM.pdf]

# Description of Additional Supplementary Files

**File name**: Supplementary Data

**Description**: Source data files containing individual data points for figure 4 and 5
