# Supplementary material for: Highly pathogenic avian influenza A(H5N1) virus in a common bottlenose dolphin (Tursiops truncatus) in Florida
Source: Commun Biol. 2024 Apr 18;7:476. doi: 10.1038/s42003-024-06173-x (PMC11026403; doi:10.1038/s42003-024-06173-x)
Supplement: Supplementary file 4 — Supplemental Data [file 42003_2024_6173_MOESM4_ESM.pdf]

Figure 4

RFU from NA-cleaved MUNANA Substrate

**A/Denmark/524/2009**	Oseltamivir Carboxylate				Zanamivir				Peramivir			
NAI Concentration (μM)	Curve 1	Curve 2	Curve 3	Curve 4	Curve 1	Curve 2	Curve 3	Curve 4	Curve 1	Curve 2	Curve 3	Curve 4
50	1445	1572	1348	1422	1478	1506	1406	1472	1545	1576	1456	1460
5	1442	1606	1414	1503	1480	1533	1486	1495	1549	1602	1580	1453
0.5	1611	1651	1516	1537	1503	1556	1475	1407	1553	1577	1498	1493
0.05	2183	2264	1866	1985	1814	1796	1724	1760	1582	1603	1518	1550
0.005	6885	6937	4437	5026	3901	3717	3557	3162	1810	1954	1669	1636
0.0005	24168	24511	15420	14924	14804	13510	10714	11719	3738	4862	3102	3164
0.00005	43565	44707	31646	31822	33805	33491	34073	33696	36098	37193	27280	27866
0.000005	46986	47540	37312	37286	36791	36463	ND	37274	44423	46028	34629	35079
0.0000005	46078	47893	38361	38598	32779	36808	35027	36054	47151	46183	36048	36083
0.00000005	46847	45788	37282	35588	36166	33021	36962	35668	45306	45624	35633	35021

**A/Denmark/528/2009**	Oseltamivir Carboxylate				Zanamivir				Peramivir			
NAI Concentration (μM)	Curve 1	Curve 2	Curve 3	Curve 4	Curve 1	Curve 2	Curve 3	Curve 4	Curve 1	Curve 2	Curve 3	Curve 4
50	1742	1739	1651	1519	1517	1508	1475	1500	1620	1637	1490	1468
5	3251	3181	2638	2827	1526	1471	1446	1392	1857	1868	1583	1620
0.5	14010	14053	10546	10749	1539	1489	1406	1476	4304	4254	3173	3084
0.05	37810	37232	31124	31213	1803	1760	1680	1756	18697	17479	13514	12391
0.005	45073	44715	38746	36575	3672	3868	3807	3641	41913	40663	32905	29883
0.0005	45522	48171	40874	40947	17743	17323	15222	15581	48831	47945	39059	37999
0.00005	46408	49559	41788	42237	35210	31610	37481	37984	46668	48699	39090	40101
0.000005	50971	51503	43909	43200	39184	39130	41637	42750	49483	49521	37869	41065
0.0000005	51072	48705	42199	43058	39249	38590	43034	42794	49834	47200	41748	41268
0.00000005	48989	48568	41491	42429	38826	37886	43409	41900	44875	46590	40485	42269

**A/common bottlenose dolphin/Florida/ UFTt2203br/2022**	Oseltamivir Carboxylate				Zanamivir				Peramivir			
NAI Concentration (μM)	Curve 1	Curve 2	Curve 3	Curve 4	Curve 1	Curve 2	Curve 3	Curve 4	Curve 1	Curve 2	Curve 3	Curve 4
50	1742	1739	1651	1519	1517	1508	1475	1500	1620	1637	1490	1468
5	3251	3181	2638	2827	1526	1471	1446	1392	1857	1868	1583	1620
0.5	14010	14053	10546	10749	1539	1489	1406	1476	4304	4254	3173	3084
0.05	37810	37232	31124	31213	1803	1760	1680	1756	18697	17479	13514	12391
0.005	45073	44715	38746	36575	3672	3868	3807	3641	41913	40663	32905	29883
0.0005	45522	48171	40874	40947	17743	17323	15222	15581	48831	47945	39059	37999
0.00005	46408	49559	41788	42237	35210	31610	37481	37984	46668	48699	39090	40101
0.000005	50971	51503	43909	43200	39184	39130	41637	42750	49483	49521	37869	41065
0.0000005	51072	48705	42199	43058	39249	38590	43034	42794	49834	47200	41748	41268
0.00000005	48989	48568	41491	42429	38826	37886	43409	41900	44875	46590	40485	42269

Figure 5

**A/common bottlenose dolphin/Florida/UFTt2203br/2022**	3'-SLN Ligand		6'-SLN Ligand	
Polymer Concentration (µg)	Replicate 1	Replicate 2	Replicate 1	Replicate 2
0.15625	0.1	0.094	0.086	0.119
0.3125	0.125	0.134	0.08	0.08
0.625	0.198	0.19	0.08	0.085
1.25	0.342	0.336	0.081	0.081
2.5	0.551	0.546	0.088	0.088
5	0.755	0.743	0.1	0.092
10	0.923	0.908	0.116	0.105
**A/bald eagle/Florida/W22-134-OP/2022**	3'-SLN Ligand		6'-SLN Ligand	
Polymer Concentration (µg)	Replicate 1	Replicate 2	Replicate 1	Replicate 2
0.15625	0.16	0.164	0.113	0.12
0.3125	0.271	0.299	0.108	0.111
0.625	0.473	0.499	0.117	0.116
1.25	0.708	0.739	0.128	0.13
2.5	0.885	0.857	0.142	0.147
5	0.971	0.989	0.175	0.189
10	1.048	0.987	0.279	0.238
**A/Memphis/257/2019**	3'-SLN Ligand		6'-SLN Ligand	
Polymer Concentration (µg)	Replicate 1	Replicate 2	Replicate 1	Replicate 2
0.15625	0.068	0.072	0.071	0.078
0.3125	0.073	0.067	0.087	0.088
0.625	0.095	0.117	0.125	0.117
1.25	0.122	0.13	0.216	0.182
2.5	0.161	0.149	0.246	0.257
5	0.188	0.183	0.38	0.361
10	0.233	0.23	0.667	0.667
